# Biological and Corrosion Evaluation of In Situ Alloyed NiTi Fabricated through Laser Powder Bed Fusion (LPBF)

**DOI:** 10.3390/ijms222413209

**Published:** 2021-12-08

**Authors:** Agnieszka Chmielewska, Anna Dobkowska, Ewa Kijeńska-Gawrońska, Michał Jakubczak, Agnieszka Krawczyńska, Emilia Choińska, Agnieszka Jastrzębska, David Dean, Bartłomiej Wysocki, Wojciech Święszkowski

**Affiliations:** 1 Faculty of Material Science and Engineering, Warsaw University of Technology, Woloska 141 Str., 02-507 Warsaw, Poland; anna.dobkowska@pw.edu.pl (A.D.); michal.jakubczak.dokt@pw.edu.pl (M.J.); agnieszka.krawczynska@pw.edu.pl (A.K.); emilia.choinska@pw.edu.pl (E.C.); agnieszka.jastrzebska@pw.edu.pl (A.J.); 2 Centre for Advanced Materials and Technologies CEZAMAT, Warsaw University of Technology, Poleczki 19 Str., 02-822 Warsaw, Poland; ewa.kijenska@pw.edu.pl; 3 Department of Plastic and Reconstructive Surgery, The Ohio State University, 915 Olentangy River Rd., Columbus, OH 43212, USA; david.dean@osumc.edu; 4 Department of Materials Science and Engineering, The Ohio State University, 140 W 19th Ave., Columbus, OH 43210, USA; 5 Centre of Digital Science and Technology, Cardinal Stefan Wyszynski University in Warsaw, Woycickiego 1/3, 01-938 Warsaw, Poland; b.wysocki@uksw.edu.pl

**Keywords:** nitinol (nickel titanium), NiTi, laser powder bed fusion (LBPF), corrosion, ion release, cytotoxicity, bacterial growth, in situ alloying

## Abstract

In this work, NiTi alloy parts were fabricated using laser powder bed fusion (LBPF) from pre-alloyed NiTi powder and in situ alloyed pure Ni and Ti powders. Comparative research on the corrosive and biological properties of both studied materials was performed. Electrochemical corrosion tests were carried out in phosphate buffered saline at 37 °C, and the degradation rate of the materials was described based on Ni ion release measurements. Cytotoxicity, bacterial growth, and adhesion to the surface of the fabricated coupons were evaluated using L929 cells and spherical *Escherichia coli* (*E. coli*) bacteria, respectively. The in situ alloyed NiTi parts exhibit slightly lower corrosion resistance in phosphate buffered saline solution than pre-alloyed NiTi. Moreover, the passive layer formed on in situ alloyed NiTi is weaker than the one formed on the NiTi fabricated from pre-alloyed NiTi powder. Furthermore, in situ alloyed NiTi and NiTi made from pre-alloyed powders have comparable cytotoxicity and biological properties. Overall, the research has shown that nitinol sintered using in situ alloyed pure Ni and Ti is potentially useful for biomedical applications.

## 1. Introduction

Personalised implants and medical instrumentation that can be adapted to the anatomy and needs of a particular patient, may bring many benefits to the healing process and improved quality of life. Additive manufacturing (AM) allows the production of personalised, complexly-shaped implants (e.g., highly curving surfaces or lattice structures), and pre-defined mechanical properties that are not possible to form using traditional manufacturing strategies [1,2,3]. Therefore, the use of AM has recently received a lot of attention from the medical industry [4]. Simultaneously, many biocompatible materials, which previously could not be used or were useful only in limited applications, have now been operationalised for AM. Besides biocompatibility, the critical issue of medical devices for skeletal reconstruction is to adjust their mechanical properties in relation to their attachment to the surrounding bone [4,5,6]. The currently predominant alloy for these devices, Ti-6Al-4V, often presents a mismatch between bone and device mechanical properties. This may lead surgically reconstructed bone to fail due to stress shielding and/or device failure due to stress concentration [7,8,9]. In the latter case, unexpected device failure is likely to require emergency revision surgery. In terms of skeletal replacement devices or fracture fixation, currently available Ti-6Al-4V hardware is associated with problems due to its high stiffness, relative to bone. NiTi alloys have been recently shown to be promising materials for reducing the risk of stress shielding and hardware failure due to their low stiffness provided by superelasticity and shape memory behaviour [10,11,12]. Moreover, the unique combination of shape memory and superelastic properties, coupled with good biocompatibility, has elevated exploration of NiTi as a candidate for medical devices. On the surface of NiTi parts, Ti ions interact with dissolved oxygen to form a passive layer of titanium dioxide (TiO_2_). That layer is extraordinarily tenacious and passive, thereby giving NiTis excellent corrosion resistance and biocompatibility. Recent studies of self-disinfecting TiO_2_ coatings have shown a high level of protection against pathogenic microbes such as *E. coli* bacteria [13,14,15]. Such surfaces eliminate microorganisms in situ and prevent the development of bacterial biofilms, which can cause degradation of NiTi 3D printed parts [16,17,18]. The TiO_2_ layer also protects NiTi materials from the external environment and limits Ni ion release, which could be hazardous for health in high local concentrations. Other researchers have reported that, although NiTi alloys have a high percentage of Ni, the quantity of Ni ions released from those alloys is minor compared with the release of toxic components from typical biocompatible materials such as stainless steel when it is used for medical application [19,20,21].

One obstacle to successful NiTi AM is the loss of Ni due to the evaporation during the laser-induced melting process. Since the evaporation temperature of Ni is lower than Ti, 2913 °C and 3287 °C, respectively, the Ni/Ti ratio shifts toward higher Ti content. The decrease of Ni/Ti ratio may increase the martensitic transformation temperature, O_2_ absorption, and formation of Ni_4_Ti_3_ precipitates [22,23,24]. For example, it was reported that each 1 at. % change in the amount of Ni results in an 80 °C shift in the transformation temperature of the alloy [25]. In addition, the Ni/Ti ratio is crucial for the medical applications of NiTi since it determines the shape memory (thermal memory) and superelastic properties of the alloy. The thermal memory properties of NiTi medical devices can be tuned to be sensitive at body temperature, which is higher than common room temperature and much higher than common refrigeration or freezing temperatures. Thus, to ensure these effects, their chemical composition must be accurately controlled. Manufacturing NiTi alloys directly from elemental powders, known as in situ alloying, if successful, would allow immediate changes in the chemical composition of the batch powder by mixing the desired amount of both alloys’ elements. Considering Ni evaporation during the fabrication process, the predetermined amount of Ni in the batch powder would likely need to be increased to obtain the desired chemical composition of the final alloy that results from laser powder-bed fusion (LPBF) part fabrication.

To date, studies on in situ alloying of NiTi have focused on the characterisation of mechanical and thermomechanical properties. To the best of the authors’ knowledge, in situ alloyed NiTi fabricated via LPBF has not been investigated in terms of corrosion resistance and biocompatibility. Hence, to the authors’ knowledge, this is the first study to characterises the microstructure, corrosion, and biocompatibility of LPBF, in situ alloyed NiTi from an elementally blended mixture of pure Ni and pure Ti powders. In this novel study, pre-alloyed NiTi powder, that has already been qualified for LPBF, was used as a control. The favourable results of this study widen the possibilities of NiTi alloy production and would have use in the validation whether NiTi alloys’ manufacturing using powders other than pre-alloyed NiTi will meet the requirements for specific biomedical applications.

## 2. Results

### 2.1. Microstructure Characterisation

**Figure 1:**
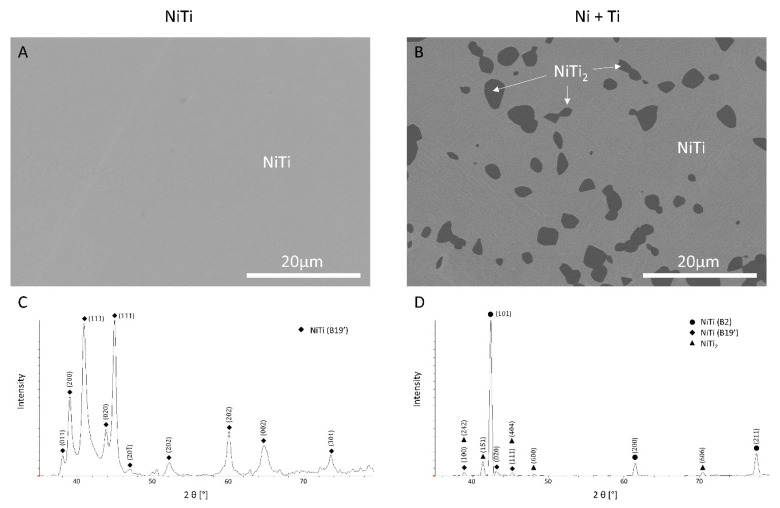
The microstructure of (**A**) NiTi and (**B**) Ni+Ti coupons under SEM BSE; XRD patterns of (**C**) NiTi and (**D**) Ni+Ti coupons.

SEM and XRD microstructure analysis of both the NiTi (i.e., alloyed prior to 3D printing) and Ni+Ti (i.e., not alloyed prior to 3D printing) coupons began with Figure 1A, where the XRD pattern depicted in Figure 1C, confirms the presence of a martensitic NiTi (B19′) phase in the pre-alloyed NiTi coupons. Despite NiTi (B19′), peaks coming from the NiTi (B2) austenite phase exist in the Ni+Ti coupons (Figure 1D). Some dark irregularly shaped phases formed in the Ni+Ti coupons (Figure 1B). As shown in Figure 1D, those phases can be identified in terms of NiTi_2_. Summing up, the microstructure of the coupon prepared from pre-alloyed NiTi powder consists of NiTi (B19′). In contrast, the microstructure of the coupon produced from the blended mixture of pure Ni and pure Ti is composed of NiTi (B19′), NiTi (B2), and NiTi_2_.

### 2.2. Surface Characterisation

**Figure 2:**
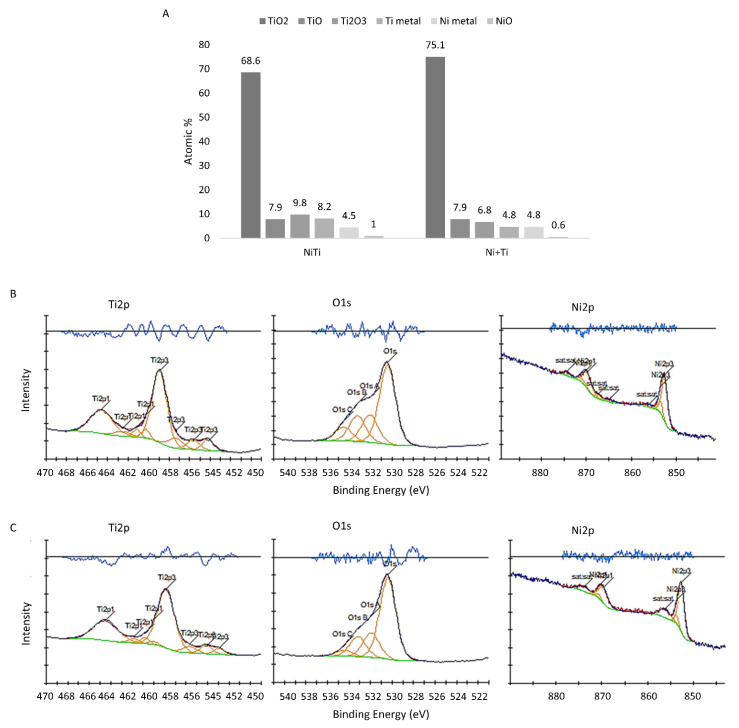
XPS results: (**A**) surface chemical state measured with XPS, (**B**) high resolution spectra of Ti 2p, O 1s and Ni 2p acquired from the NiTi coupon surface, (**C**) high resolution spectra of Ti 2p, O 1s and Ni 2p acquired from the Ni+Ti coupon surface.

Figure 2A shows the phase chemical state of the top layer of NiTi and Ni+Ti coupons. The XPS spectra corresponding to NiTi and Ni+Ti coupons are presented in Figure 2B and Figure 2C, respectively. The dominant oxide on the surface of both coupons is TiO_2_. Nevertheless, a fraction of TiO and TiO_3_ titanium oxides, as well as Ti in pure a metallic state, were observed. Moreover, 1% and 0.6% of NiO was detected on the surface of the NiTi and Ni+Ti coupons, respectively, as was the presence of 4.5 and 4.8% of a pure Ni metallic state, respectively.

### 2.3. Corrosion Behaviour

**Figure 3:**
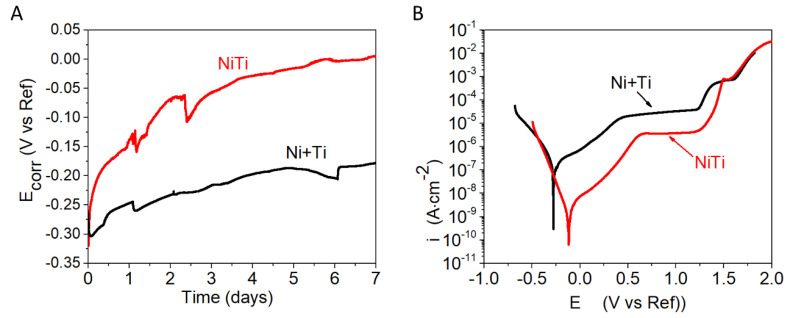
The results delivered from the electrochemical measurements performed in PBS: (**A**) Ecorr evaluation, (**B**) potentiodynamic polarisation curves.

Figure 3A depicts the evolution of the E_corr_ with time acquired from the NiTi and Ni+Ti coupons suspended in PBS solution. Values of E_corr_ obtained for Ni+Ti are more negative than those observed for the NiTi coupon, suggesting more corrosion of the Ni+Ti alloy occurred in this corrosive medium [26]. A rapid rise of the E_corr_ value is observed for the NiTi coupons, starting from −0.32 V/Ref approaching O V/Ref. The E_corr_ value observed for Ni+Ti decreased abruptly at the beginning of immersion from −0.23 V/Ref to −0.30 V/Ref. Afterward, it has started to increase approaching −0.20 V/Ref at the end of immersion time.

Potentiodynamic polarisation curves observed for both coupons are shown in Figure 3B. The characteristics for potentiodynamic measurements parameters are given in Table 1. The higher corrosion current density (i_corr_ = 130 nA∙cm^−2^) and more positive values of corrosion potential (E_corr_ = −0.13 V/Ref) were calculated and are shown for coupons made from pre-alloyed NiTi powder. Lower values of E_corr_ and higher i_corr_ were observed for the Ni+Ti material. Both curves exhibit primarily two regions in their anodic branches, namely an active region at lower anodic potentials and a passive region at higher anodic potentials (indicated by the current plateau). The maximum current density at the active region is higher for the Ni+Ti coupon than for the NiTi coupon. This can be attributed to variation in the rate of the metal oxidation [27,28]. Next, the protective oxide layer was formed over the surface of both alloys, and a sudden increase of current density is observed due to its breakdown (pitting potential, E_pit_, for both is similar and it is found to be −1.28 V/Ref for NiTi and −1.24 V/Ref for Ni+Ti, Table 1).

**Figure 4:**
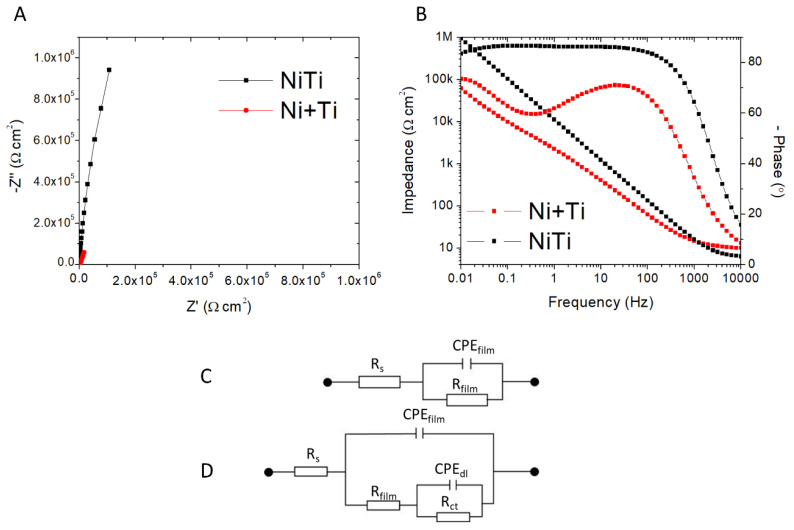
EIS data recorded for the analysed coupons presented in the form of (**A**) Nyquist plots and (**B**) Bode plots; (**C**) equivalent circuit used for fitting the EIS results obtained for NiTi, and (**D**) equivalent circuit used for fitting the Electrochemical Impedance Spectroscopy (EIS) results obtained for Ni+Ti.

EIS (Electrochemical Impedance Spectroscopy) data for NiTi and Ni+Ti taken after 7 days of immersion in PBS are presented in Figure 4. Although the shape of the Nyquist plots are similar, there are distinguishable differences between the diameters of the semicircles. The semicircle observed for the pre-alloyed NiTi coupon is wider, indicating enhancement of polarization resistance. As shown in Figure 4B, one time constant can be recognized for NiTi, whilst the Bode plot observed for the Ni+Ti presents two different time constants. Therefore, two various equivalent circuits have been chosen to fit the EIS data (Figure 4C,D). For the NiTi coupons, a simple Randles cell was used to fit the data [29]. In this circuit, R_s_ is an electrolyte resistance, R_film_ is the resistance of the oxide film. To account for the nonideal capacitive behaviour of the oxide, constant-phase element CPE_film_ was chosen [30,31,32]. The EIS data obtained for the Ni+Ti coupons can be fitted employing the equivalent circuit model, as shown in Figure 4D, with two time constants indicating the presence of the oxide film. The oxide film is composed of two layers: the porous oxide layer, that can be penetrated by the electrolyte, and a second layer closer to the unoxidised metal. In this model, R_S_ is also solution resistance, CPE_film_ is the constant phase element of the passive film layer, and CPE_dl_ is the double-layer capacitance. The passive film resistance, as described by R_film_, and R_ct_, represents charge transfer resistance [27,33,34].

The diameter of the capacitive loop in the Nyquist plots shows that both materials exhibit good corrosion resistance (Figure 4A) [27]. The high absolute value of the impedance at low frequency, and a phase angle close to 90°, are typical capacitive behaviour for nitinol immersed in PBS (Figure 4B) [35,36]. The fitting parameters are given in Table 2. The material produced from pre-alloyed NiTi powder exhibits the highest total resistance value. This indicates that the corrosion resistance of this alloy is higher than the corrosion resistance of Ni+Ti. Additionally, the greater the value of R_film_, better corrosion resistance of the film. According to that observation, the oxide film formed on the NiTi coupon is more resistant to corrosion than the film formed on the Ni+Ti coupon. The R_film_ for the NiTi coupon is about 20 MΩ cm^2^, whereas R_film_ for Ni+Ti is only 7 kΩ cm^2^.

**Figure 5:**
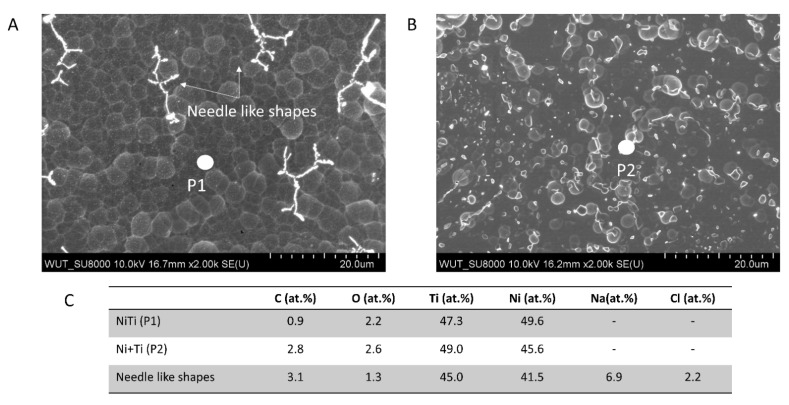
Surface morphology of the coupons after 7 days of immersion in PBS under open-circuit conditions: (**A**) NiTi, (**B**) Ni+Ti, (**C**) EDX point analysis made in P1 and P2.

SEM observations before and after immersion in PBS were performed to have additional data on the corrosion behaviour of the analysed materials (Figure 5). The NiTi surface is observed to be uniformly covered with an oxide film and needle-like shapes (Figure 5A). The EDX point analysis indicates that those shapes may be identified with NaCl crystals (Figure 5C) [37]. The Ni+Ti coupon was also covered with oxide film as well as globular nodules that are distributed non-uniformly across the entire surface after immersion (Figure 5B). The EDX chemical analyses did not reveal any Ca or P. This suggests that after 7 days of immersion in PBS, hydroxyapatite growth does not occur on either type of coupons (Figure 5C).

**Figure 6:**
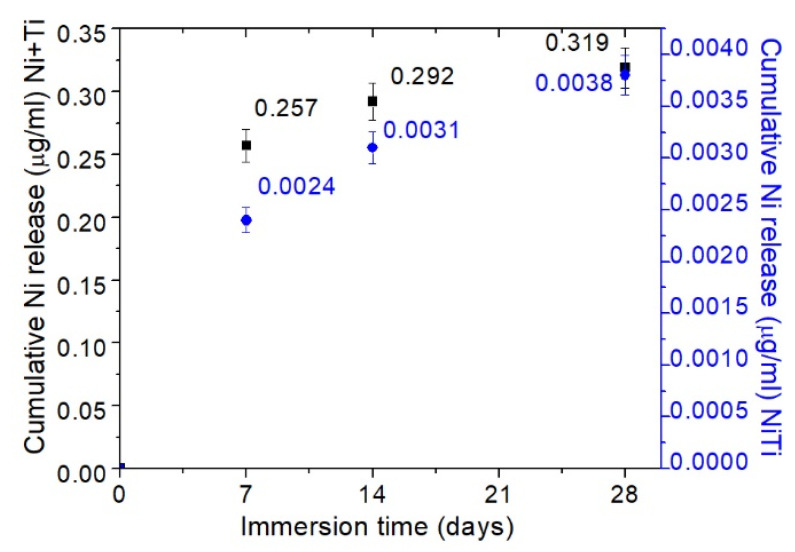
Ni ion release from NiTi (blue) and Ni+Ti (black) coupons as a function of immersion time.

Ni ion release during immersion in PBS was measured to describe the corrosion behaviour of both coupons more thoroughly as well as potential biocompatibility issues. Cumulative amounts of Ni ion release in PBS must be studied as a function of immersion time as presented in Figure 6. In the case of both materials, the largest amount of nickel ion release occurs during the first 7 days. Thereafter, the rate and amount of Ni ion release slows down. After 7 days of immersion of NiTi coupons, 0.0024 µg/mL of Ni was found in the solution versus 0.257 µg/mL of Ni was observed in solution following a 7 day immersion of Ni+Ti coupons. The observed difference may confirm the electrochemical results. The electrochemical results suggest that Ni+Ti is more corrosive (i.e., offers less resistance to corrosion) than NiTi in PBS. When the time of immersion was extended to 28 days, Ni+Ti coupons continued the trend of higher Ni ion release than NiTi; however, that that difference was no longer statistically significant. Note that the cumulative Ni ion release after the first 7 days of Ni+Ti coupon immersion was observed to be more than 100 times greater than the NiTi coupons. Thereafter, the difference between Ni ion release in the two types of coupons decreased. After 14 days, around 90 times more Ni ions were released from Ni+Ti coupons than from the NiTi coupons. After 28 days of immersion the difference also decreased, and cumulative Ni release was around 80 times higher for the Ni+Ti coupons than for the NiTi coupons.

### 2.4. Biological Properties

**Figure 7:**
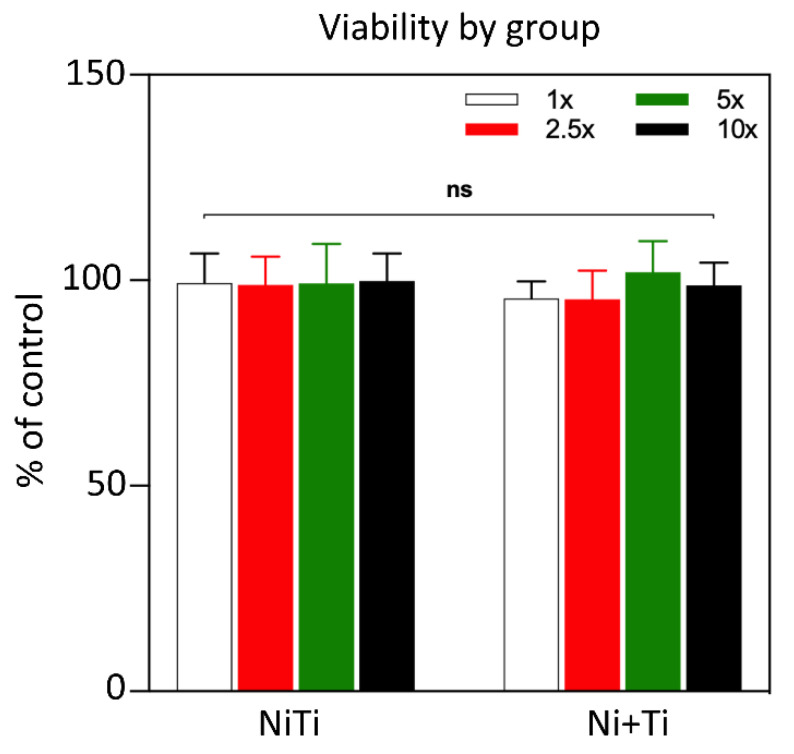
Cytotoxicity (% viability) of L929 fibroblast cell line treated with NiTi or Ni+Ti solute aliquots (1× [i.e., undiluted], 2.5×, 5× and 10× indicate dilution factor of the solutes).

Figure 7 shows the viability of L929 cells treated with NiTi or Ni+Ti solute (i.e., recovered immersion media) for 24h. The viability of the cells cultured in non-diluted solute compared to control was 99.4% and 95.5% for NiTi and Ni+Ti, respectively. The cell viability results obtained for 2.5, 5, and 10× diluted media of both tested materials were close to those for non-diluted solute and were within the range of 95.5–102% (the differences are not significant).

**Figure 8:**
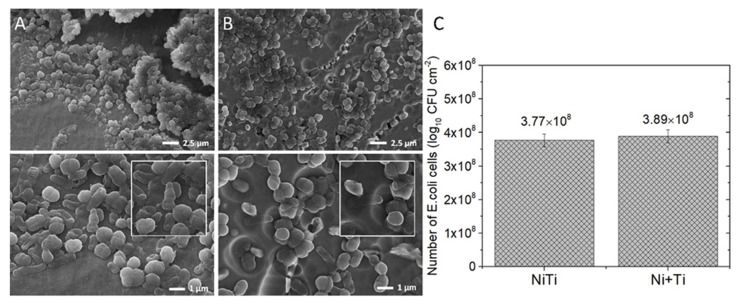
SEM images of E. coli adhesion to the surface of: (**A**) NiTi and (**B**) Ni+Ti coupons; (**C**) the number of bacteria cells washed out from the surface after biofilm formation.

The SEM images of *E. coli*-generated biofilm that developed on the NiTi and Ni+Ti coupons are presented in Figure 8A and Figure 8B, respectively. Figure 8C depicts the number of *E. coli* cells recovered after washing the NiTi, and Ni+Ti unalloyed coupons. The surfaces of investigated coupons are covered with a large number of bacterial cells, which is additionally confirmed by the results obtained from the pour plate method (Figure 8C). The results shown were observed uniformly across each coupon’s surface, with no preferential sights of biofilm formation or cell death. The morphology of the cells on both types of coupons is similar and the cells retain their spherical (viable) form [38]. In addition, the spherical *E. coli* bacteria did not adhere firmly to the surface and were easily washed off the investigated coupons. As calculated, the surfaces of NiTi and Ni+Ti alloy coupons were well-coated, with 3.77 ± 1.23 × 10^8^ and 3.89 ± 2.12 × 10^8^ CFU cells per cm^2^, respectively (Figure 8C).

## 3. Discussion

In this study, the corrosion and biological properties of NiTi pre-alloyed and Ni+Ti unalloyed powders used in LPBF manufacturing of test coupons with an Ni:Ti ratio of 55.7:44.3 (at. %) were studied. The form of batch powders used during LBPF determined the microstructure and phase composition of the alloys the LPBF-printed coupons. The parts fabricated from pre-alloyed NiTi powder presented the NiTi (B19′) martensitic phase. In contrast, assays of the parts prepared by LPBF from blended elementally pure (i.e., unalloyed) Ni and Ti powders presented NiTi (B19′), NiTi (B2), and NiTi_2_.

Chemical polishing of both types of coupons in HF:HNO_3_ removed particles that were sintered to the surface. Loosely sintered powder particles are expected following LPBF fabrication [39,40]. Moreover, it is known that the chemical processing of NiTi alloys is an efficient method, not only for the elimination of unmelted, loosely sintered particles and other small surface defects, but also as a way of improving biomedically desirable surface oxidation [41,42]. The qualitative chemical state of both coupons surface, characterised with XPS was observed to be identical; however, the quantitative results differed between the two types of coupons. Titanium oxide TiO_2_ is a dominant phase, and the amount observed is 68.6 and 75.1% for the NiTi and Ni+Ti coupons, respectively. Moreover, the fraction of TiO and TiO_3_ titanium oxides, as well as Ti in a pure metallic state, was determined. Ni in a pure metallic state and NiO oxide found on the surfaces may suppress the release of Ni ions.

The corrosion potential of NiTi produced from elementally blended pure Ni and Ti experienced a pronounced decline compared to coupons prepared from pre-alloyed NiTi powder, presented a reduction in the corrosion resistance of the Ni+Ti coupons. The fact that the Ni+Ti specimen is more corrosive in PBS is confirmed by the numerical values of corrosion current in the range of which the potentiodynamic polarisation curve for that coupon was observed. Both coupons exhibit a tendency for passivation. However, for Ni+Ti, passivity occurs in a slightly wider range of potentials, suggesting a lower tendency for localised corrosion versus the coupons prepared from pre-alloyed NiTi.

The more active behaviour of the material fabricated from a mixture of pure Ni and pure Ti in PBS is also confirmed by Ni ion release and EIS results. More Ni ions were released from the alloy prepared using the elementally blended powders than from the alloy produced from the pre-alloyed powder. The amount of released Ni ions is related to the stability and thickness of the oxide film formed on the surface, which was previously shown by Shabalovskaya et al. [43] and Nasakina et al. [44]. Three factors are expected to suppress the leaching of Ni ions from the coupons produced using a mixture of pure Ni and pure Ti during the immersion. First, the higher amount of metallic Ni found via XPS on the surface of the coupons prepared from elementally blended pure Ni and pure Ti, can effectively impede Ni ion release during the entire immersion time. Second, the amount of released Ni ions is dependent on the crystal structure of phases formed in the materials. As shown in this work, the NiTi phase in coupons prepared from pre-alloyed NiTi powder is present only in the form of monoclinic B19′, whereas both monoclinic B19′ and cubic B2 NiTi phases, as well as a NiTi_2_ phase, exist in coupons fabricated from the elementally blended mixture of pure Ni and Ti. The surface energy values of dense surface configurations with a stoichiometric ratio for B2-NiTi (101) and B19’-NiTi (010) are 1.81 J/m^2^ and 1.93 J/m^2^, respectively [45], which can reduce Ni ion release. Third, corrosion processes are microstructure-dependent and the presence of NiTi_2_ supports micro-galvanic corrosion between the matrix and secondary phases (NiTi behaves as cathode, whilst NiTi_2_ is an anode) [46].

The cytotoxicity of both materials appears to be minor and similar. The materials were found to demonstrate more than 95.5% viability at all dilution factors, and the differences between the percentage of the viability of all tested dilutions of both groups of coupons are not significant. According to the ISO 10993-5 cytotoxicity testing standard, which states that any material reducing the cell number by equal to or more than 30% is toxic. Neither of the two groups of coupons tested have a cytotoxic effect on L929 cells. A similar situation is observed for bacterial growth and bacterial adhesion to the surface of both types of coupons. Similar cytotoxicity properties were observed for both with no significant differences between both types of coupons. TiO_2_-coated surfaces may reduce bacterial adhesion due to the alternation of surface free energy [47]. It is assumed that bacterial adhesion occurs with the least intensity on low-energy surfaces because of weaker binding at the interface [48]. However, Zhang et al. [47] have shown that bacterial adhesion may be minimal at specific optimal surface energies, all of which are due to multiple factors. According to Derjaguin–Landau–Verwey–Overbeek’s (DLVO) theory, bacteria adhere to the surface when the summary of all possible interactions gives a negative surface energy value. As all interactions are related to the distance of separation, bacterial cells adhere after they are in close contact with the surface. Thus, at close contact, *E. coli* bacteria encounter a deep-primary energy minima and, therefore, adhere [49,50]. As TiO_2_ coating increases surface energy, the bacterial adhesion would be modulated (reduced) [13,51].

It must be noted that Ni ion release from both types of coupons studied is much lower than what is observed for the conventionally manufactured NiTi alloy investigated by Meng et al. [52]. Moreover, the NiTi coupons prepared from elementally blended pure Ni and Ti exhibited lower corrosion resistance than the coupons LPBF fabricated from pre-alloyed NiTi powder. Results presented in this work show opportunities for further investigation of elementally pure Ni and Ti powders as a substitute for pre-alloyed NiTi powder. This is confirmed by the similar cytotoxicity and similar biological behaviour of both the pre-alloyed NiTi and coupons prepared from elementally pure Ni+Ti. In future studies it may be possible to improve the corrosion properties of the coupon produced from a mixture of pure Ni and Ti elements. Further research is also needed, especially on heat treatments, that could result in a single phase microstructure of NiTi alloy coupons rendered from elementally pure Ni and pure Ti source materials.

## 4. Materials and Methods

### 4.1. Materials and Fabrication Procedure

Pre-alloyed Ni_55.7_Ti_44.3_ powder, referred to as NiTi, and elementally pure Ni and pure Ti powders, referred to as Ni+Ti, blended in the ratio Ni:Ti = 55.7:44.3 wt %, were used to fabricate materials using LPBF. All powders were provided by TLS Technik GmbH (Bitterfeld-Wolfen, Germany) and had a spherical shape and particle size below 63 µm. The chemical compositions of powders (including impurities) are shown in Table 3. A Realizer SLM50 machine (Realizer GmbH, Borchen, Germany) was employed for LPBF fabrication of cylindrical coupons with a dimension of ϕ = 5 mm. LPBF manufacturing was carried out under an Ar atmosphere with an O_2_ concentration below 0.3%. A two-step melting-remelting process was applied. The first step, referred to as melting, was used to consolidate the powder. Various laser process parameters were applied to the second step, referred to as remelting, to improve final part density. The parameters of both the melting and the remelting steps used for manufacturing NiTi and Ni+Ti parts are given in Table 4. The manufacturing procedure has been previously described in detail [53]. To homogenise chemical and phase composition, Ni+Ti materials were subjected to a solution heat treatment composed of a two-step vacuum heat treatment performed at 900 °C for 24 h followed by solutionising at 1150 °C for another 24 h and subsequent immediate quenching in water. All parts were ultrasonically cleaned three times in deionised water for 15 min, and then chemically polished in a solution composed of 7.5 HF: 50 HNO_3_: 42.5 H_2_O to remove particles sintered to the surface. The chemical polishing procedure has been previously described in detail [39].

### 4.2. Microstructural Characterisation

The microstructure of the coupons was examined using a Hitachi (Tokyo, Japan) SU8000 scanning electron microscope (SEM) in backscattered electron mode (BSE To undertake this examination metallographic samples were prepared. Coupons were polished on Saphire 550 grinding and polishing machine (ATM Qness GmbH, Germany) using SiC papers (from #600 to #4000, 15 min each), and subsequently polished using 0.1 µm alumina oxide suspensions for 30 min. Concurrent rotation of grinder and head, equal to 120 min^−1^, was used in each step. The single pressing force applied to the sample was 10N. Afterward, coupons were polished by an Ar beam using a Hitachi IM4000 (Tokyo, Japan) ion milling system. The phase composition was identified at room temperature using a Bruker (Billerica, MA, USA) X-ray diffraction (XRD) device. Filtered Cu Kα (λ = 0.154056 nm) radiation was used at operating values of 40 kV and 40 mA. The XRD data were collected over 2Θ angular range of 35–80°, with a step Δ2Θ–0.05° and count time of 3 s.

### 4.3. Surface Chemical State Characterisation

The surface chemical state of the coupons was characterised using a Thermo Electron Corporation (Waltham, MA) Microlab 350 XPS spectrometer. A monochromatised Al *Ka* radiation source with 300 W power and energy of 1486.6 eV was used. Measurements were carried out in the vacuum range of ~10^−9^ mbar. To determine the chemical states and concentrations of Ti 2p, O 1s, and Ni 2p, high-resolution spectra were acquired at a pass energy of 40 eV at a 0.1 eV/step. The C 1s peak was used to correct binding energy positions.

### 4.4. Corrosion Behaviour

#### 4.4.1. Electrochemical Procedure

To determine corrosion behaviour of both alloys in the simulated body fluid solutions, electrochemical measurements were carried out in naturally aerated, quiescent PBS solution at 37 °C. The measurements were carried out using a FAS1 Gamry potentiostat equipped with three electrodes: platinum as the counter electrode, Ag/AgCl as the reference electrode, and the measured sample was the working electrode. The reference electrode was inserted into a Luggin capillary. The setup was placed in front of the working electrode. Phosphate buffered saline (PBS) solution (pH 7.4) was prepared by adding a Sigma Aldrich (St. Louis, MO, USA) certified tablet to 200 mL of distilled. The corrosion potential (E_corr_) of the coupons was recorded for 7 days under open-circuit conditions. Then, the potentiodynamic polarisation tests were carried out after immersion in the range of 0.5 V below E_OCP_ to 2 V vs. E_OCP_ (a scan rate of 5 mV/s was used). The corrosion potential (E_corr_) and current density (i_corr_) were calculated by the Tafel extrapolation method [54]. At least three tests were conducted for each specimen. Electrochemical Impedance Spectroscopy (EIS) measurements were recorded just after immersion of the coupons in PBS solution. The EIS tests were performed in a frequency range from 0.01 to 10,000 Hz with a sinusoidal signal amplitude of 10 mV. The polarisation curves were fitted using Gamry’s (Philadelphia, PA, USA) Elchem software in Tafel mode. EIS data were also fitted using Gamry Elchem Software. SEM surface observations were made before and after immersion in PBS under open-circuit conditions to better understand the corrosion processes.

#### 4.4.2. Ni Ion Release

Ni ion release evaluation of the coupons was performed according to ASTM F3306 [55]. Each coupon was placed in a separate container filled with PBS that prepared according to the ASTM F2129 [56] protocol. A coupon surface area to PBS volume ratio of 0.1 cm^2^/mL was used, as recommended by the US Food and Drug Administration (FDA) (Silver Spring, MD, USA). All containers were placed in a heating chamber at 37 ± 2 °C for 7, 14, and 21 days. At each time point, NiTi and Ni+Ti coupon were removed from the solution and the immersion solutes for Ni ion measurement were collected. NiTi and Ni+Ti coupons were immediately transferred into a new container filled with fresh PBS solution. The relative concentration of Ni ions was measured using inductively coupled plasma mass spectrometry (ICP-MS) on a Perkin Elmer (Waltham, MA, USA) PE Elan DRC-e machine.

### 4.5. Biological Behaviour

#### 4.5.1. Cytotoxicity Study

The cytotoxicity of the coupons was evaluated as per the ISO 10993-5 standard [57,58,59]. The analysis began with the proliferation of American Type Culture Collection (ATCC) (Manassas, VA, USA) L929 murine fibroblast cells in a complete medium of Dulbecco’s Modified Eagle Medium (DMEM) supplemented with heat-inactivated fetal bovine serum (FBS) and 1% penicillin-streptomycin in 5% CO_2_. The cells were proliferated in an incubator at 37 °C for 2–3 days until sufficient confluence was obtained. Next, the cells were detached with 0.05% trypsin-EDTA and seeded in 96-well plates at a concentration of 10,000/well in 100 µL of media. Meanwhile, coupons were sterilised by dipping in 70% ethanol solution, followed by 1h exposure to UV, and three rinses with PBS. Solutes were obtained by soaking both types of coupons (*n* = 5) in a complete cell culture medium, including DMEM, 10% FBS, and 1% PS. The mass of the material in the volume of the extraction vehicle was adjusted to 200 mg/mL (according to ISO 10993-12 standard) [60]. These specimens were held in solution for 72 h in an incubator (37 °C, 5% CO_2_). The 72 h solutes were utilised in four dilutions (1×, 2.5×, 5×, and 10×) and each dilution was prepared in triplicate. After removing cell culture media, 100 µL of each solute was added to a well where cells were then incubated for 24 h. Thereafter, release media were aspirated from each well, and replaced with 100 μL fresh DMEM supplemented with 1% penicillin and 20 μL MTS solution and incubated for 2 h at 37 °C (5% CO_2_). After 2 h, aliquots were transferred to fresh 96-well plates and the absorbance was read at 490 nm using a microplate reader (FLUOstar Omega BMG Labtech, Offenburg, Germany). Cell viability in each the solutes was reported as the percentage of viable cells that grew in complete medium under the same conditions (i.e., the “negative control”).

#### 4.5.2. Bacterial Growth and Adhesion to the Surface

Bacterial growth and adhesion to the surface of the coupons was evaluated using ATCC 10799 spherical *E. coli* bacteria [61,62,63]. Bacteria cells were cultivated on Merck (Readington Township, NJ, USA) nutrient agar for 24 h at 37 °C. Then, the cells were harvested from a solid medium and resuspended in PBS containing 10 g L^−1^ of glucose. The final density of bacterial suspension was adjusted to 1.09 × 10^9^ CFU mL^−1^. Further, NiTi and Ni+Ti disc-shaped coupons were placed in 24-well plates and topped with 2 mL of the bacterial suspension. The suspended coupons were incubated for 24 h at 37 °C. After incubation, the coupons were washed with 3 mL and transferred into 9 mL of PBS. The number of bacterial cells in the obtained suspension was determined using a pour plate method. The results were calculated in relation to the surface area of the coupons. Biofilm formed on the coupons was observed using a Hitachi SU8000 SEM. To approach this, NiTi and Ni+Ti coupons were immersed in 3 mL of glutaraldehyde (3% *v*/*v*) solution and kept overnight. Then, the NiTi and Ni+Ti coupons were washed with PBS and dehydrated using the increasing concentrations of ethanol. Prior to SEM imaging, the coupons were coated with a thin layer of gold using a BAL-TEC Corporation (Canonsburg, PA, USA) SCD 005 sputter coater.

## 5. Conclusions

This study evaluated the corrosion and biological performance of NiTi alloy fabricated by LPBF using two different source materials with the same initial chemical composition: pre-alloyed NiTi powder and elementally blended Ni and Ti powders. It was observed that:

- The corrosion resistance of LPBF-printed parts produced from elementally blended pure Ni and Ti is slightly lower when compared to parts which were LPBF-printed from pre-alloyed NiTi powder; however, both materials show similar biocompatibility in terms of cytotoxicity.
- The low cytotoxicity and high passivation suggest that elementally blended pure Ni and Ti powders merit further investigation for use in biomedical applications.
- Further research on the LPBF manufacturing and post-printing process parameters and microstructure-dependent corrosion of elementally pure Ni and pure Ti is merited given the potential cost savings and improvements in corrosion and biocompatibility behaviour.

## Figures and Tables

**Table 1:** Electrochemical Parameters. The electrochemical parameters were calculated from Tafel extrapolation based on results from potentiodynamic polarisation tests recorded in phosphate buffered saline (PBS).

Materials	E_corr_ (V/Ref)	i_corr_ (nA∙cm^−2^)	E_b_ (V/Ref)
NiTi	−0.13	130	−1.28
Ni+Ti	−0.27	201	−1.24

**Table 2:** Equivalent circuit parameters obtained from Electrochemical Impedance Spectroscopy (EIS) data.

	R_s_ (Ω cm^2^)	R_film_ (Ω cm^2^)	CPE_film_ (µF cm^−2^)	n_1_	R_ct_ (Ω cm^2^)	CPE_dl_ (µF∙cm^−2^)	n_2_
NiTi	6	20 × 10^6^	15	0.96	-	-	-
Ni+Ti	8	7 × 10^3^	79	0.83	9 × 10^6^	90	0.82

**Table 3:** The chemical composition of powders used (from the powder manufacturer).

Material	Elements (wt.%)						
	Ti	Ni	C	O	N	H	Fe
NiTi	bal.	55.7	0.005	0.046	0.007	0.0006	
Ti	bal.		0.01	0.13	0.1	0.001	0.11
Ni		99.9	0.017				<0.1

**Table 4:** LPBF manufacturing parameters (NiTi is pre-alloyed and Ni+Ti is in situ-alloyed material).

		Laser Power P [W]	Scanning Speed v [mm/s]	Hatch Distance h [µm]
NiTi	melting	108	100	120
	remelting	22	500	120
Ni+Ti	melting	30	500	30
	remelting	25	500	30

## Data Availability

Not applicable.
